# The use of partially slatted floor designs as an alternative to littered systems in broiler chickens. II. The effects on welfare and behavioral traits

**DOI:** 10.1007/s11250-026-05149-9

**Published:** 2026-06-26

**Authors:** Hatice Çavdarcı, Musa Sarıca, Kadir Erensoy, Resul Aslan, Numan Karaçay

**Affiliations:** 1 Ladik Vocational School, Ondokuz Mayıs University, Ladik, Samsun, 55760 Türkiye; 2 Department of Animal Science, Agricultural Faculty, Ondokuz Mayıs University, Atakum, Samsun 55139 Türkiye

**Keywords:** Slatted floors, Litters, Welfare, Behavior, Broilers

## Abstract

This study investigated the effects of different flooring designs on the welfare and behavioral traits in fast-growing broilers. A total of 600 day-old Ross-308 chickens were randomly allocated to five treatment groups: fully littered (FL), fully slatted (FS), and combined systems with varying slatted floor ratios (1/3SF, 1/2SF, and 2/3SF). Welfare parameters, including body defects, gait score, and tonic immobility (TI), along with behavioral activities, were assessed. Results indicated that hock burn and breast skin burn scores were significantly higher in male birds and increased with the proportion of slatted flooring (*p* < 0.001). Conversely, the FS and high-ratio slatted groups exhibited significantly better body cleanliness and breast feathering scores compared to the FL group. While gait scores were generally poorer in males, the floor design significantly influenced walking ability (*p* = 0.05), with higher impairment observed in FS males. TI duration did not differ significantly among treatments. Behavioral observations revealed that the FL system promoted active behaviors, particularly foraging and locomotion (*p* < 0.001). In contrast, the FS system inhibited natural behaviors such as dust bathing and foraging, leading to increased preening and passive behaviors. In conclusion, while slatted floor systems offers advantages in hygiene and cleanliness, it negatively impacts leg health and restricts the expression of natural behaviors. Partially slatted floor systems presented intermediate results but did not fully mitigate the welfare trade-offs associated with slatted systems.

## Introduction

Poultry meat production has been steadily increasing for many years (Sarıca et al. 2024). In modern broiler production, management practices and housing conditions play a critical role in determining not only performance but also animal welfare and behavioral traits (Bessei 2018; Abd El-Wahab et al. 2020). Since broiler chickens spend their entire lives on a floor substrate, the type and characteristics of the flooring are highly important. To the extent that litter quality can be maintained, normal behavioral characteristics and animal welfare are maximized (Homidon and Robertson 2003; Mohammed et al. 2019; Sánchez-Casanova et al. 2020). While cage and slatted floor systems can also be used, the litter system is the most common worldwide (Altan and 2024; Sarıca and Erensoy 2020).

Maintaining litter quality for an extended period becomes increasingly difficult at higher stocking densities, and high litter moisture often leads to severe welfare issues, such as contact dermatitis (Petek et al. 2014; Da Costa et al. 2014). Cage and slatted floor systems have been considered as alternatives, but they raise concerns regarding limited movement and negative impacts on leg-breast health and behavioral traits (Shields and Greger 2013). Feet and leg problems are particularly important indicators of welfare. Broilers spend up to 76% of their time lying down, making the floor substrate critical to their physical comfort and walking ability (Weeks et al. 2000). The absence of a friable substrate, such as litter, in fully slatted systems restricts essential natural behaviors. For example, broilers can exhibit most natural behaviors, such as scratching, foraging, and dust bathing, when in contact with litter (Villagra et al. 2014; Baxter et al. 2018). In contrast, quantifying these behavioral shifts, Ghayas et al. (2021) observed that resting behavior reached up to 60% in intensive indoor systems compared to 45% in free-range setups with better outdoor access.

In slatted flooring systems, manure accumulating under the slats is separated from the birds. Since chickens do not come into contact with manure, footpad dermatitis, hock, and breast lesions/burns caused by litter can be minimized (Topal 2021; Sonnabend et al. 2022). Almeida et al. (2017) and Li et al. (2017) demonstrated significant improvements in feather cleanliness and a reduction in breast burns when birds were reared on slatted floors rather than littered. However, replacing litter entirely with hard slatted materials introduces other physical stressors. Dikmen and Gündüz (2025) reported that rearing broilers on fully slatted floors increased hock burns (score 1.30 vs 1.20) and footpad dermatitis (score 2.60 vs 0.95) compared to cage systems, while also restricting behaviors such as dust bathing and foraging (Baxter et al. 2018; Chuppava et al. 2018).

Although there are numerous studies evaluating broilers’ welfare characteristics on different flooring materials, generalized comparisons are no longer sufficient to understand specific trade-offs. Previous studies have provided specific findings; for example, Kaukonen et al. (2017) evaluated the effects of elevated platforms on contact dermatitis, Chuppava et al. (2018) investigated footpad health across distinct floor designs, and Çavuşoğlu and Petek (2019) quantified the specific behavioral differences between birds on slatted versus littered floors. Despite these specific investigations, studies focusing specifically on plastic slats and the combined use of both systems remain scarce (Sonnabend et al. 2022; May et al. 2024).

While the first series of this comprehensive experiment (Aslan et al. 2024) focused primarily on growth, performance, carcass characteristics, and litter moisture, the present continuation study aims to address a distinct knowledge gap. Specifically, it was hypothesized that welfare and behavioral impairments that may arise from the use of fully littered or slatted systems could be mitigated by combining them. Therefore, this study aimed to determine the specific effects of fully littered, fully slatted, and different partially slatted combinations (1/3 slatted flooring + 2/3 litter; 1/2 slatted flooring + 1/2 litter; 2/3 slatted flooring + 1/3 litter) on the physical welfare and behavioral traits of fast-growing broilers. In this way, the possibility of a production system with lower stress, higher welfare standards, and greater expression of natural behaviors in sustainable broiler production was investigated.

## Material and methods

**Fig. 1:**
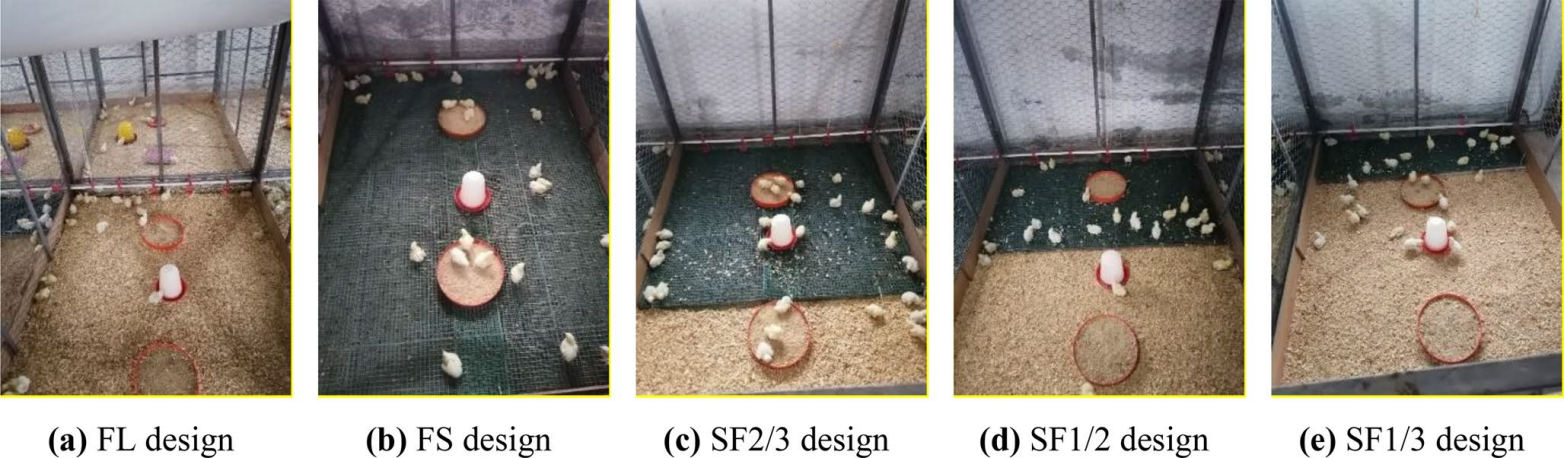
Fully littered (FL, **a**), fully slatted (FS, **b**) and partially slatted (**c**: 1/3 littered+2/3 slatted: 2/3SF, **d**: ½ littered + ½ slatted: 1/2SF, **e**: 2/3 littered+1/3 slatted: 1/3SF) floor designs (Aslan et al. 2024)

This study was conducted at Ondokuz Mayıs University, Agricultural Faculty, Research and Application Farm. The experiment was carried out in an environmentally controlled poultry house and experimental pens with dimensions of 1.8 × 2.0 m (pen floor area: 3.6 m^2^) and a height of 1.5 m, separated by a wire mesh. In the study, five treatment groups were formed as fully littered (FL), fully slatted (FS), 1/3 littered+2/3 (2/3 FS), 1/2 littered+1/2 slatted (1/2 SF), 2/3 littered+1/3 slatted (1/3 SF). The slats were specially manufactured for poultry, made of durable plastic, with a height of 10 cm and grid spacing measuring 1.5 cm (Fig. 1).

The experiment was initiated with 600 fast-growing broiler chickens (Ross 308) mixed male-female at day old age and lasted for 6 weeks. A total of 120 day-old chicks were randomly allocated to 5 replicates (24 chicks each, 6.8 birds/m^2^) with equal numbers of males and females in each floor treatment group.

Wood shavings (8–10 cm thick, 4 kg/m^2^) were used in the littered pens. In the slatted pens, the floor was covered with plastic netting during the first week to facilitate movement. The birds had ad libitum access to feed and water throughout the experiment. They were fed according to a four-phase commercial feed program: chick starter feed (23% CP, 3000 kcal/kg ME) from days 1–10, broiler chick feed (22% CP, 3100 kcal/kg ME) from days 11–21, broiler feed (22% CP, 3100 kcal/kg ME), days 22–35 (21% CP, 3100 kcal/kg ME), and finisher (18% CP, 3100 kcal/kg ME) 36–42 days. The house temperature was gradually reduced from 33 to 34 °C on the first day to 21 °C by the fourth week. Lighting was provided for 24 h during the first 3 days, 20 h during the next 2 weeks, and 18 h during the 4–6 weeks. The birds were vaccinated against Newcastle (at 1, 11, and 22 days) and Gumboro disease (at 16 days). This continuation study focuses on the effects of these flooring systems on specific welfare and behavioral characteristics, while the results related to growth, performance, and carcass traits were evaluated in the first series of this study (Aslan et al. 2024).

### Welfare parameters

**Fig. 2:**
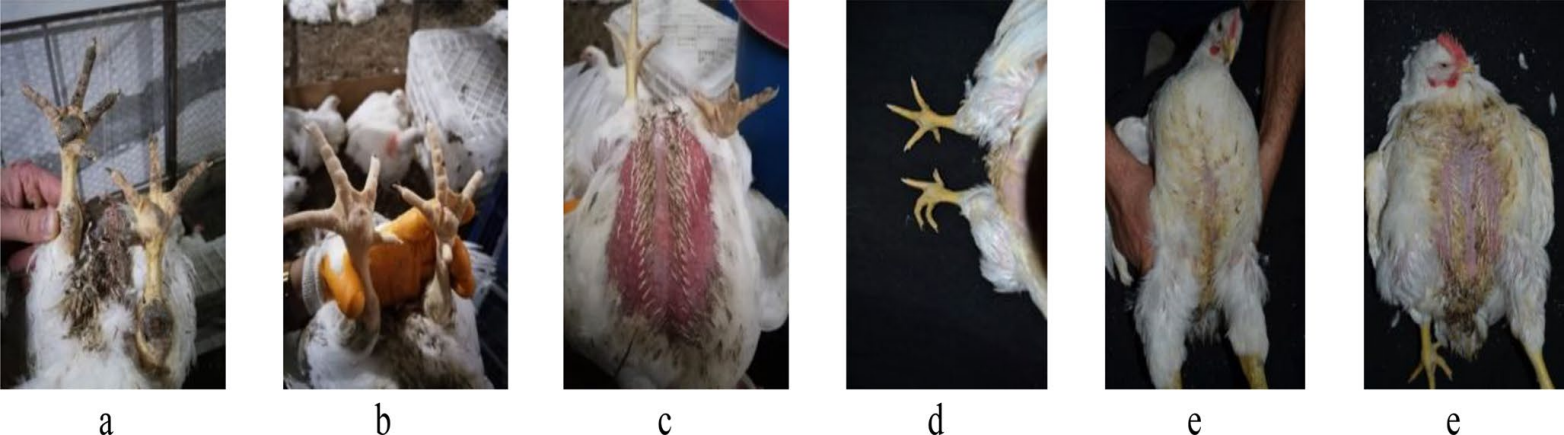
Images of some body defects in broilers (**a**: hock burn, **b**: foot-pad dermatitis, **c**: breast burn, **d**: crooked toes, **e**: breast feathering level)

*Body defects.* Footpad dermatitis (FPD) (Welfare Quality 2009), hock burn (HB) (Welfare Quality 2009), breast skin burn or discoloration (BB) (Erensoy and Sarıca 2023), crooked toes (CT) (Erensoy and Sarıca 2023), body cleanliness (BC) (Welfare Quality 2009), and breast feathering (BF) (Welfare Quality 2009) scores were determined. A total of 120 broilers (24 birds per treatment group) were assessed at 42 days of age. All welfare and behavioral evaluations throughout the experiment were performed by the same trained and experienced observer to ensure consistency (details are presented in Table 1 and Fig. 2).

**Table 1:** Procedures for determining welfare indicators

Score	FPD/HB	BB	CT	BC	BF
0	No lesions or color changes	No deformation	All toes are intact and straight	The entire body is clean	Completely covered with feathers
1	Spot lesions and color changes exist	Slight redness	1–2 toes crooked	Feet, legs, and feathers are 25% dirty	Slight feather loss
2	Lesions exist on 50% of the foot-pad/hock	Moderate redness	3–4 toes crooked	Feet, legs, and feathers are 50% dirty	Moderate feather loss
3	Lesions exist on 75% of the foot-pad/hock	Severe redness	5–6 toes crooked	Feet, legs, and feathers are 75% dirty	Severe feather loss
4	Severe lesions, swelling, or bleeding present on the foot-pad/hock	Severe lesion, swelling, or bledding exist on breast skin	7–8 toes crooked	Feet, legs, and feathers are completely dirty	Completely featherless breast

FPD: Foot-pad dermatitis, HB: Hock burn, BB: Breast burn/discoloration, CT: Crooked toes, BC: Body cleanliness, BF: Breast feathering level

*Gait score.* A gait score assessment test was performed on 3 female and 3 male chicks aged 42 days, randomly selected from each batch. The walking score was performed for all chickens by the same observer, following the 0–5 scoring system developed by Kestin et al. (1992). A score of 0 indicates no abnormality in walking; 1 indicates the chick walks irregularly, taking unusually long steps; 2 indicates a diagnosable level of leg impairment, which does not prevent access to feed and water; 3 indicates the bird shows lameness or angulation (ie., valgus-varus) of the leg and has a tendency to collapse when forced to move; 4 indicates severe walking impairment in the chick, which can only walk with assistance; a score of 5 indicates that the bird cannot walk and attempts to crawl.

**Fig. 3:**
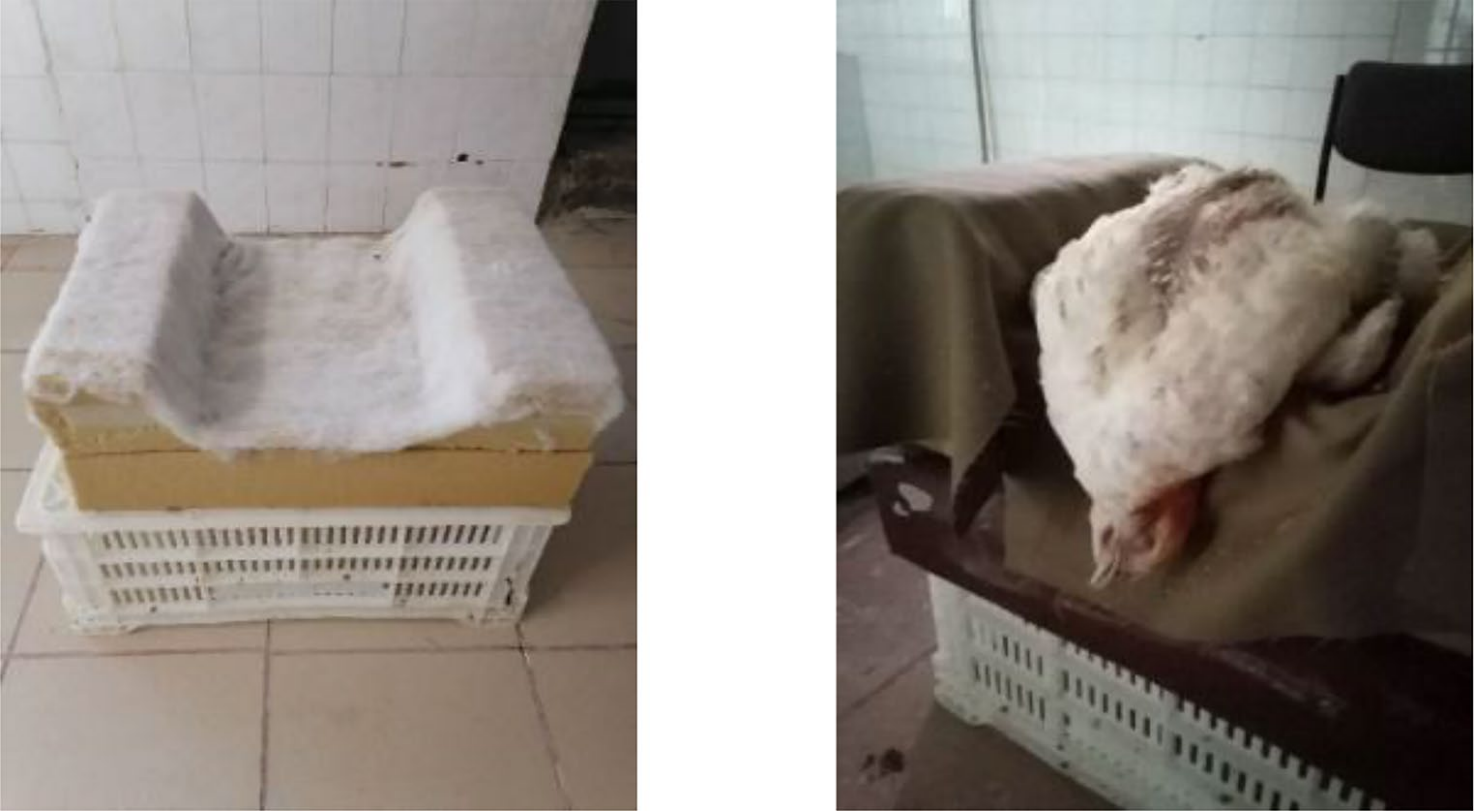
U-shaped soft bedding platform used for the tonic immobility (TI) test

*Tonic immobility (TI) test.* The TI test was performed individually on one bird at a time, selecting 3 male and 3 female birds from each replicate at 42 days of age (15 males and 15 females for each treatment). To avoid interaction effects from prolonged holding times and repeated handling, birds were handled gently and tested immediately. The selected bird was placed in a separate, dark room and laid on their backs in a soft, U-shaped cradle (as shown in Fig. 3). The cradle was positioned horizontally without any incline to standardize the procedure. The bird’s head was oriented downwards and its breast was gently pressed for 15 sec. After being released, birds that remained motionless for 10 sec were considered to have exhibited TI. The time until the bird turned onto its right side or stood up was recorded by an observer standing 1 m away as the TI duration (in sec). If TI did not happen in 5 repeated attempts, a score of 0 was given. The maximum test duration was limited to 600 sec. The number of inductions required to achieve TI was also recorded for each chicken (Prieto and Campo 2010; Sarıca et al. 2025).

*Behavioral observations.* Behavioral traits were classified as passive (resting, sleeping), active (standing, locomotion, feather pecking, aggressiveness, running, exploratory, foraging, eating, drinking), and comfort behaviors (preening, stretching, dust bathing). Behaviour was observed at pen level using instantaneous scan sampling at six ages (day 6, 13, 20, 27, 34, and 41). Video recordings were taken at 3 different time intervals (9–10 a.m., 1–2 p.m., 5–6 p.m.) on each observation day using video cameras. The behavior of the birds in the pen was determined by instantaneous sampling every 20 minutes for each time interval. This means that a total of 21 scans were performed for each pen on each observation day. In each scan, the behaviors of all broilers in the pen were determined according to the ethogram in Table 2. Behavioral observations were performed by the same observer. The number of birds exhibiting specific behaviors in each pen was divided by the total number of birds and expressed as the percentage of the respective behavior (Fortomaris et al. 2007; Sarıca et al. 2007; Boz et al. 2021).

**Table 2:** Ethogram used for behavioural observations

Category	Behavior	Expression of the behavior
Passive	Resting	Sitting or lying down on the litter without any other behavior
	Sleeping	Sitting or lying down on the litter with eyes closed
Active	Standing	Standing still without doing any activity on its feet
	Locomotion	Walking around without doing other activities
	Feather pecking	Gently pecking and pulling the feathers of other birds in the pen
	Aggressiveness	Jumping on each other, threatening from the front, walking aggressively towards each other, jumping in place, aggressively pecking at other birds’ heads, kicking or flapping wings
	Escape	Avoiding any stimulus, being alert, crouching, or freezing
	Exploratory	Pecking the pen’s accessories and equipment
	Foraging	Pecking at the ground, searching for food by scratching with the feet
	Eating	Eating feed from the feeder
	Drinking	Drinking water from a nipple drinker
Comfort	Preening	Playing with their own feathers and preening
	Stretching	Stretching by extending one leg and one wing
	Dust bathing	Cleaning the feathers by rubbing the body with the head and body using the litter material, mixing the litter with the feet, and trying to insert the litter material between the feathers with upright wing movement

*Statistical Analyses.* Different floor treatments in broiler chickens were evaluated according to the randomized block design. One-way analysis of variance was applied in the statistical analysis of behavioral characteristics. When differences between means were significant (*p* < 0.05), differences between means were determined using the Tukey’s multiple comparison test. All data were tested for normality using Kolmogorov-Smirnov test. When data did not show a normal distribution, a transformation appropriate for the data type was first applied, and normality was retested. For behavioral data expressed as percentages that did not show a normal distribution, a transformation (arcsin transformation) was performed, and statistical analyses were conducted. However, the actual means were used in the interpretation of the traits. If the data still did not follow a normal distribution after the transformation, the Mann Whitney-U and Kruskal-Wallis tests were used. In the statistical model, the pen was considered the experimental unit for behavioral observations, while the individual bird was considered the observational unit for welfare and physical parameters. SPSS 21.0 software package was used for statistical analyses.

## Results

*Welfare traits.* No signs of FPD were observed in any of the five treatment groups consisting of fully littered (FL), fully slatted (FS), and littered-slatted combinations, with similar results seen across all groups.

**Fig. 4:**
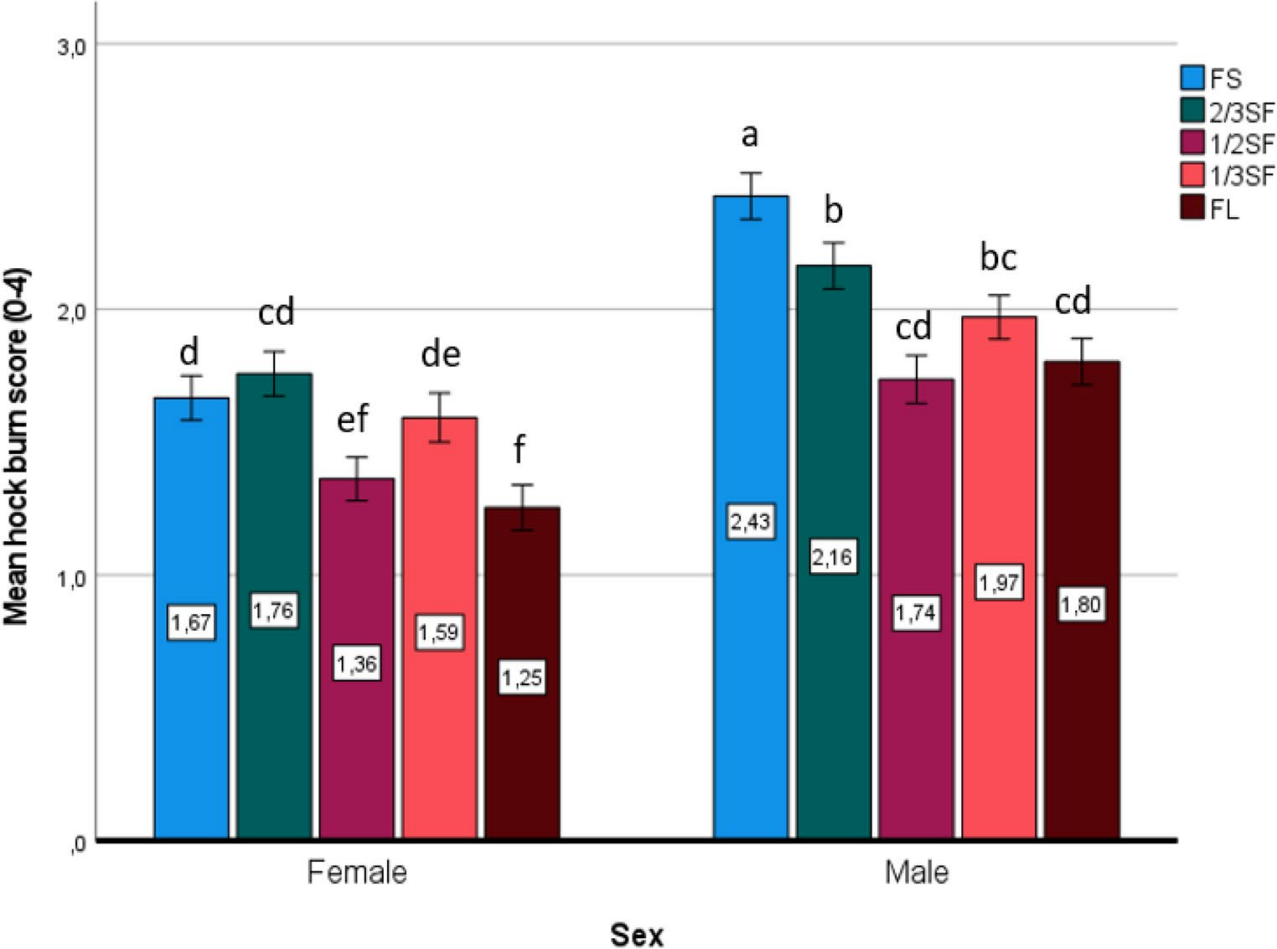
The effects of different flooring systems on hock burn scores in broilers. Kruskal-Wallis *χ*^*2*^ = 41.214; *df=*4; *p*_*(treatment)*_*<*0.001; Kruskal-Wallis *χ*^*2*^ = 32.944; *df=*1; *p*_*(sex)*_*<*0.001; Kruskal-Wallis *χ*^*2*^ = 89.419; *df=*9; *p*_*(treatment × sex)*_*<*0.001. FS: fully slatted system, 2/3SF: 2/3 slatted + 1/3 littered system, 1/2SF: 1/2 slatted + 1/2 littered system, 1/3SF: 1/3 slatted + 2/3 littered system, FL: fully littered system. ^a-f^: means indicated by different letters are significantly different based on the Tukey-HSD test results at the *p* < 0.05 level

The effect of different flooring systems, sex, and the interaction between flooring type and sex on HB scores was found statistically significant (*p* < 0.001), as shown in Fig. 4. Male chickens’ HB scores (1.74–2.43) were higher than those of females (1.25–1.76) in all groups. Among the experimental groups, the highest HB score of 2.43 was observed in males reared in the FS system, while the lowest score of 1.25 was recorded in females in the FL group. The FS and 2/3SF groups, which had a high slatted ratio in males, were statistically different (*p* < 0.05) from the other combined and littered systems; while in females, the highest HB score was determined in the 2/3SF (1.76) group. In general, it was determined that as the slatted flooring area increased, the HB scores increased in both sex, and as the littered area ratio increased, the lesion severity decreased.

**Fig. 5:**
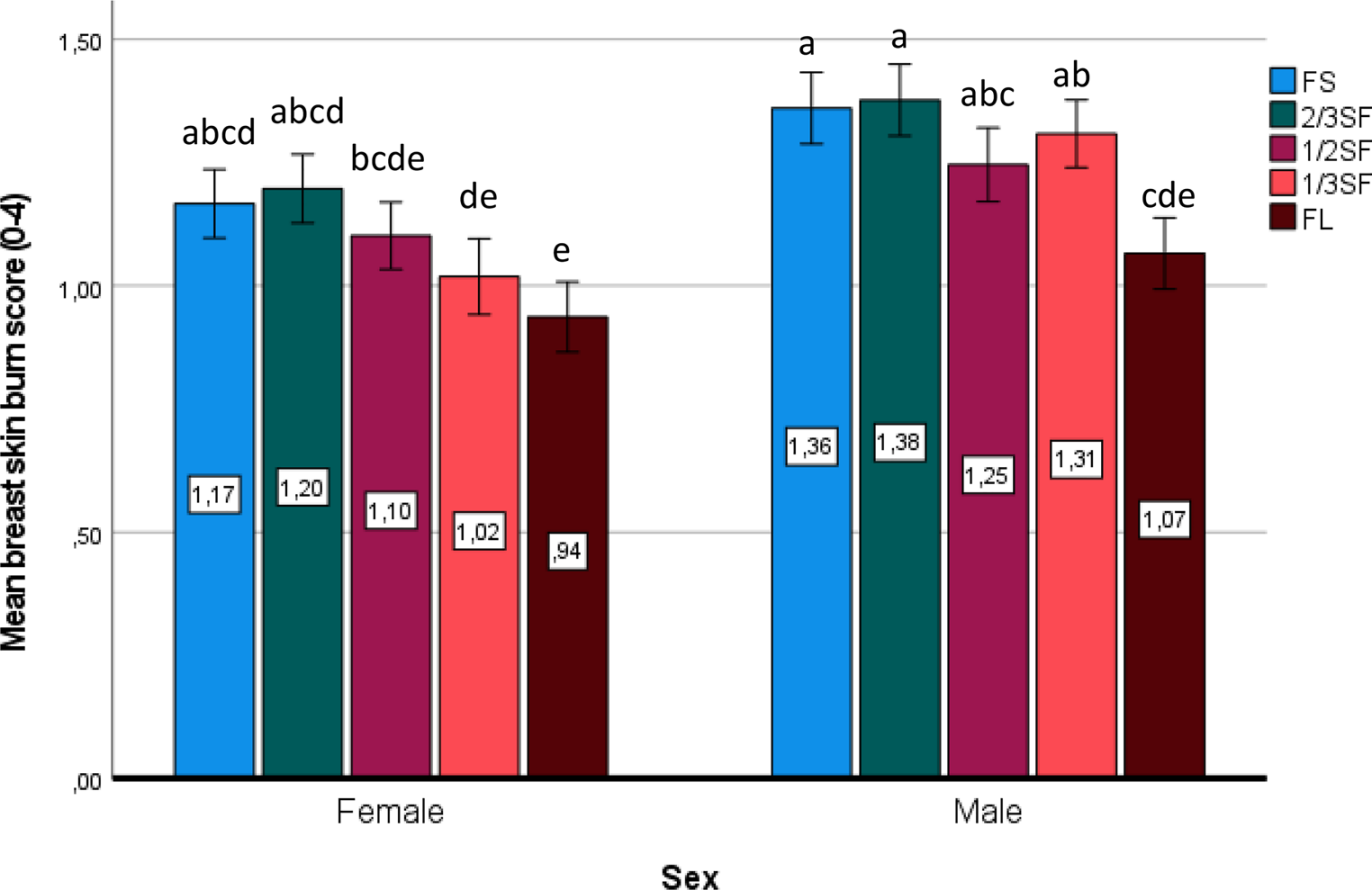
The effects of different flooring systems on breast skin burn scores in broilers. Kruskal-Wallis *χ*^*2*^ = 43.743; *df=*4; *p*_*(treatment)*_*<*0.001; Kruskal-Wallis *χ*^*2*^ = 13.319; *df=*1; *p*_*(sex)*_*<*0.001; Kruskal-Wallis *χ*^*2*^ = 63.576; *df=*9; *p*_*(treatment × sex)*_*<*0.001. FS: fully slatted system, 2/3SF: 2/3 slatted + 1/3 littered system, 1/2SF: 1/2 slatted + 1/2 littered system, 1/3SF: 1/3 slatted + 2/3 littered system, FL: fully littered system. ^a-e^: means indicated by different letters are significantly different based on the Tukey-HSD test results at the *p* < 0.05 level

The effect of different floor designs on breast skin burn/color change score in broiler chickens is illustrated in Fig. 5. Treatment (*χ2* = 43.743; df = 4; *p* < 0.001), sex (*χ2* = 13.319; df = 1; *p* < 0.001), and their interaction (*χ2* = 63.576; df = 9; *p* < 0.001) were found to have a statistically significant effect on the breast color change score. Male chickens had higher mean scores than females in all groups. In female birds, the highest score was 1.20 in the 2/3SF group, while the lowest score was 0.94 in the FL group. In male birds, the highest averages were recorded in the 2/3SF (1.38) and FS (1.36) groups, respectively, while the lowest average was observed in the FL group at 1.07.

The effects of different flooring designs on crooked toes in broiler chickens were insignificant. Toe deformities were observed in only one bird each in the FL and FS groups; no such deformities were observed in the other groups.

**Fig. 6:**
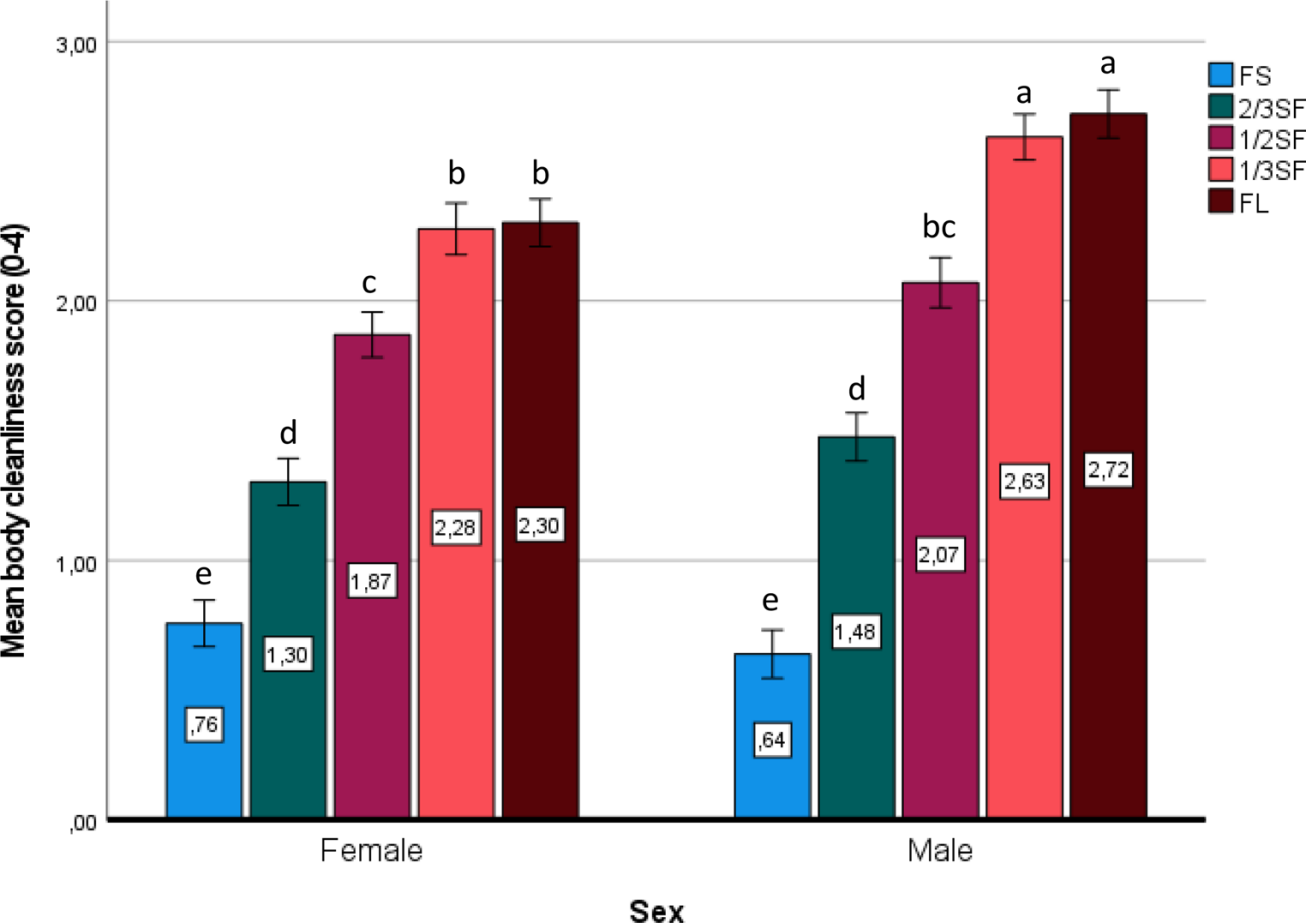
The effects of different flooring systems on body cleanliness scores in broilers. Kruskal-Wallis *χ*^*2*^ = 142.911; *df=*4; *p*_*(treatment)*_*<*0.001; Kruskal-Wallis *χ*^*2*^ = 9.875; *df=*1; *p*_*(sex)*_*=*0.002; Kruskal-Wallis *χ*^*2*^ = 155.859; *df=*9; *p*_*(treatment × sex)*_*<*0.001. FS: fully slatted system, 2/3SF: 2/3 slatted + 1/3 littered system, 1/2SF: 1/2 slatted + 1/2 littered system, 1/3SF: 1/3 slatted + 2/3 littered system, FL: fully littered system. ^a-e^: means indicated by different letters are significantly different based on the Tukey-HSD test results at the *p* < 0.05 level

The effects of different floor designs on body cleanliness scores in broiler chickens is shown in Fig. 6. Treatment (χ2 = 142.911; df = 4; *p* < 0.001), sex (χ2 = 9.875; df = 1; *p* = 0.002), and their interaction (χ2 = 155.859; df = 9; *p* < 0.001) were found to have a statistically significant effect on body cleanliness score. In female chickens, the highest score was statistically similar in the FL (2.30) and 1/3SF (2.28) groups, while the lowest score was found in the FS group (0.76). In males, the highest averages were again recorded in the statistically non-differentiated FL (2.72) and 1/3SF (2.63) groups, while the lowest average was determined in the FS group with 0.64.

**Fig. 7:**
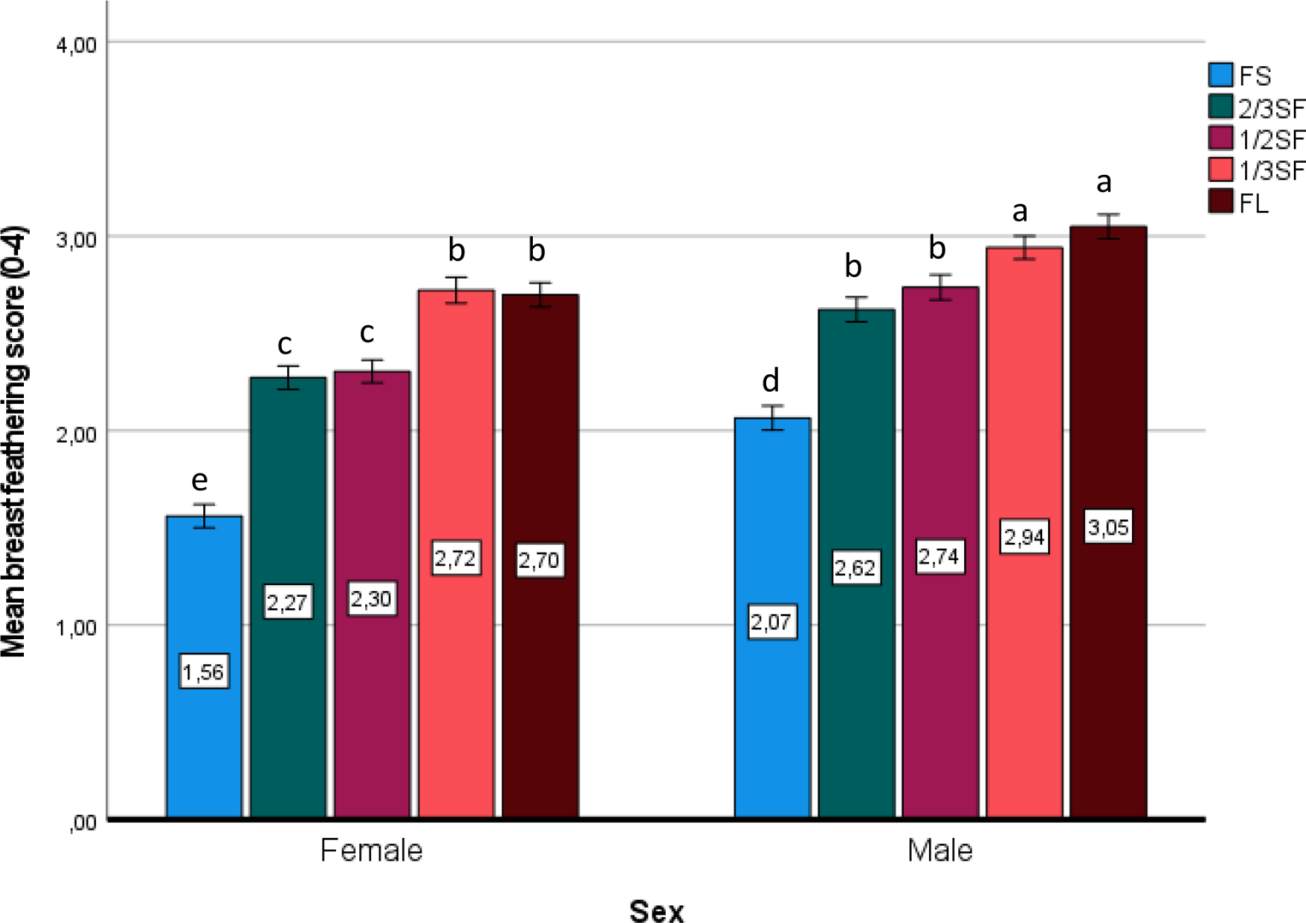
The effects of different flooring systems on breast feathering score in broilers. Kruskal-Wallis *χ*^*2*^ = 17.507; *df=*4; *p*_*(treatment)*_*=*0.002; Kruskal-Wallis *χ*^*2*^ = 6.435; *df=*1; *p*_*(sex)*_*=*0.011; Kruskal-Wallis *χ*^*2*^ = 43.337; *df=*9; *p*_*(treatment × sex)*_*<*0.001. FS: fully slatted system, 2/3SF: 2/3 slatted + 1/3 littered system, 1/2SF: 1/2 slatted + 1/2 littered system, 1/3SF: 1/3 slatted + 2/3 littered system, FL: fully littered system. ^a-e^: means indicated by different letters are significantly different based on the Tukey-HSD test results at the *p* < 0.05 level

The effects of different floor designs on breast feathering score in broilers is given in Fig. 7. The treatment (χ2 = 17.507; df = 4; *p* = 0.002), sex (χ2 = 6.435; df = 1; *p* = 0.011), and their interaction (χ2 = 43.337; df = 9; *p* < 0.001) were significant effect on the breast feathering score. In female chickens, the lowest score was observed in the FS group with an average of 1.56, while the highest scores were found in the 1/3SF (2.72) and FL (2.70) groups. Similarly, in male birds, the lowest score was 2.07 in the FS group; the highest averages were recorded in the statistically non-significant FL (3.05) and 1/3SF (2.94) groups.

**Fig. 8:**
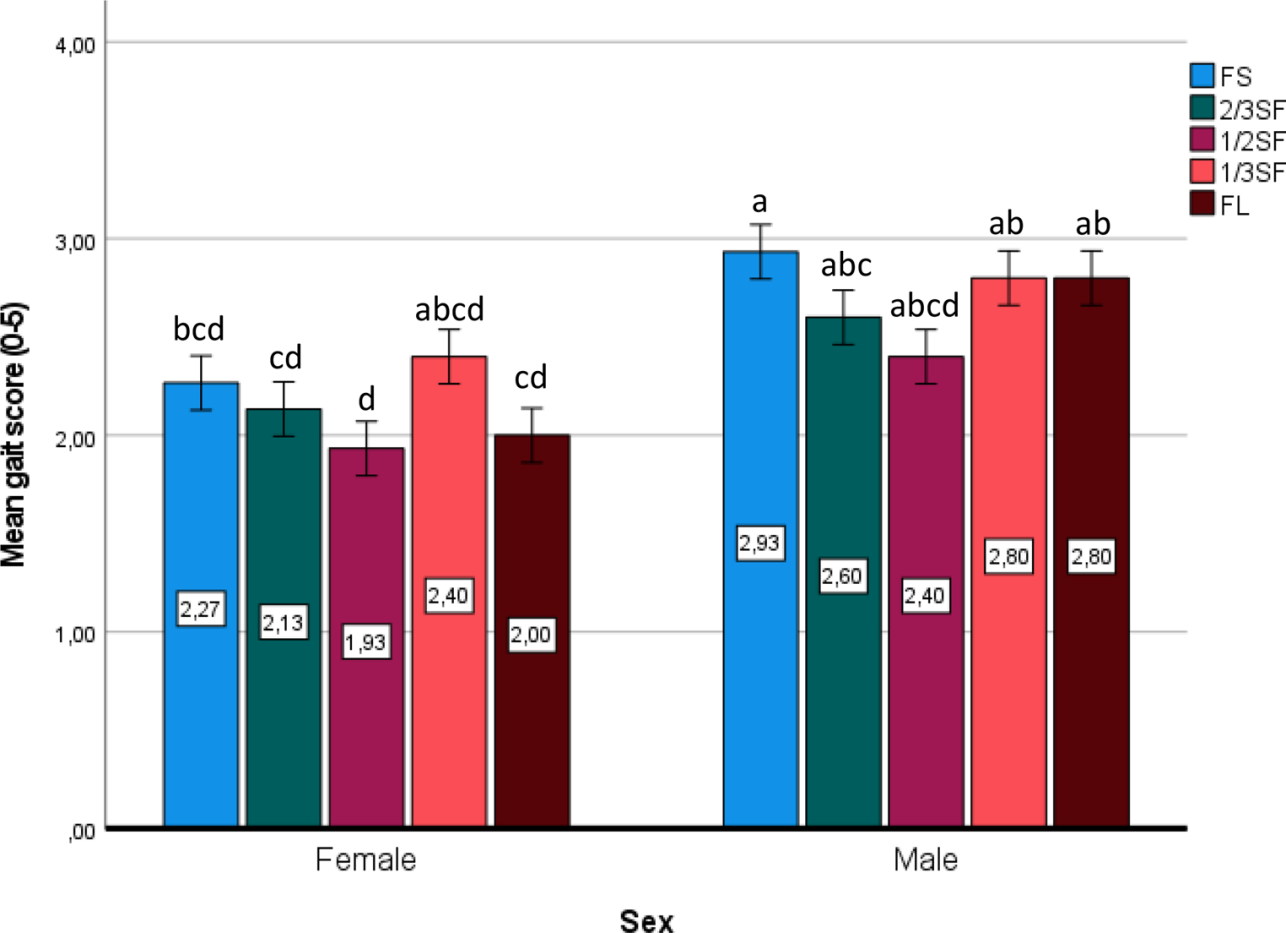
The effects of different flooring systems on the gait scores in broilers. Kruskal-Wallis *χ*^*2*^ = 9.650; *df=*4; *p*_*(treatment)*_*=*0.05; Kruskal-Wallis *χ*^*2*^ = 32.067; *df=*1; *p*_*(sex)*_*<*0.01; Kruskal- Wallis *χ*^*2*^*=* 48.822; *df=*9; *p*_(*treatment× sex)*_*<*0.01. FS: fully slatted system, 2/3SF: 2/3 slatted + 1/3 littered system, 1/2SF: 1/2 slatted + 1/2 littered system, 1/3SF: 1/3 slatted + 2/3 littered system, FL: fully littered system. ^a-d^: means indicated by different letters are significantly different based on the Tukey-HSD test results at the *p* < 0.05 level

The effects of different floor designs on the gait score in broiler chickens is illustrated in Fig. 8. The treatment (χ2 = 9.650; *p* = 0.05), sex (χ2 = 32.067; *p* < 0.01) and their interaction (χ2 = 48.822; *p* < 0.01) were found statistically significant. The interaction effect revealed that, in general, male birds had higher mean walking scores than females. Among all groups, the highest walking score was found in the male FS group with a mean of 2.93, followed by the male 1/3SF and male FL groups with a mean of 2.80. In females, the highest score was recorded in the 1/3SF group with 2.40, and the lowest score was recorded in the 1/2SF group with 1.93. The lowest average in male chicks was determined to be 2.40 in the 1/2SF group, which was equal to the highest value in the female group.

**Fig. 9:**
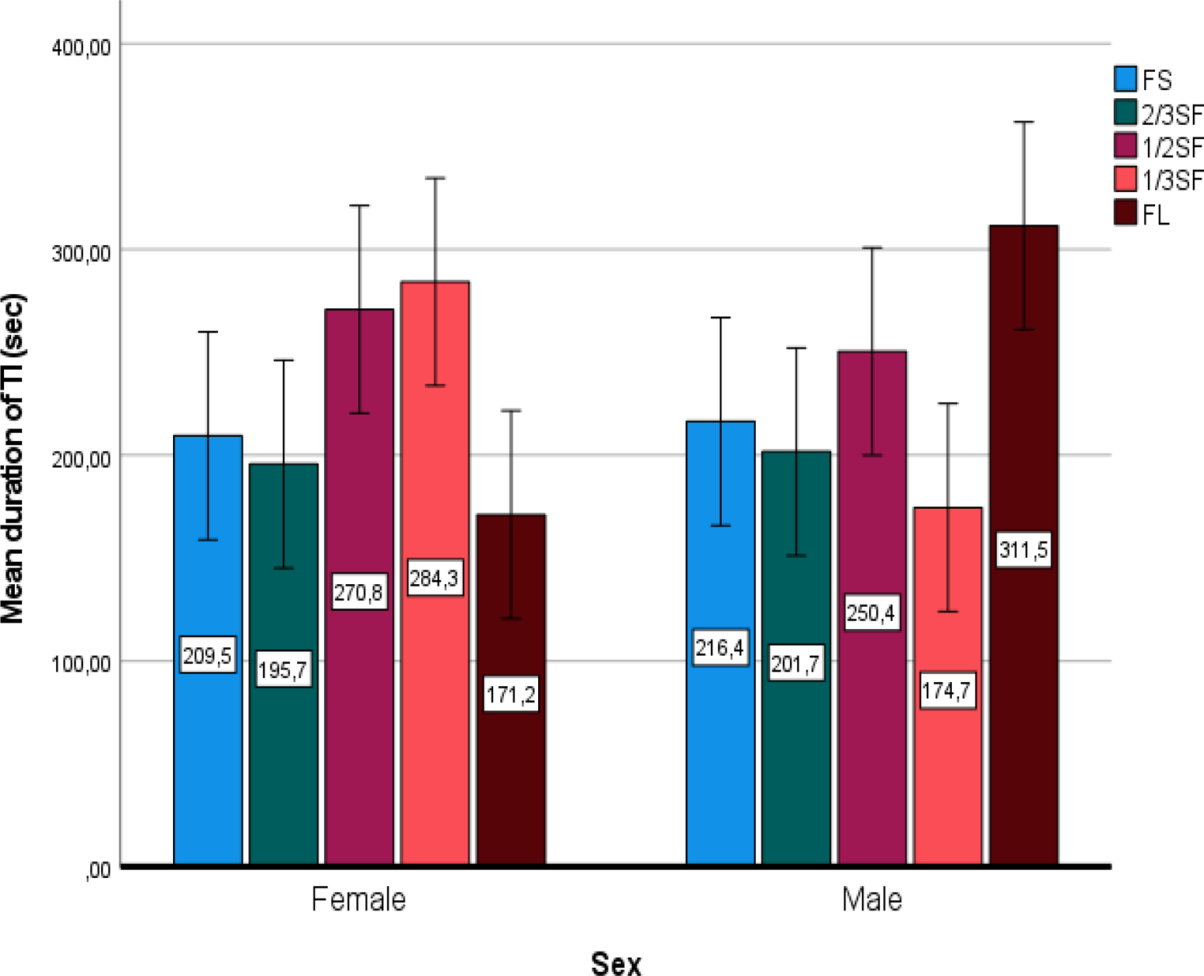
The effects of different flooring systems on the duration of TI in broilers. *F*(4,149) = 0.43, *p*_*(treatment)*_*=*0.788; *F*(1,149) = 0.12, *p*_*(sex)*_*=*0.726; *F*(4,149) = 0.57, *p*_*(treatment × sex)*_*=*0.684. FS: fully slatted system, 2/3SF: 2/3 slatted + 1/3 littered system, 1/2SF: 1/2 slatted + 1/2 littered system, 1/3SF: 1/3 slatted + 2/3 littered system, FL: fully littered system

The effects of different floor designs on the duration of tonic immobility (TI) in broiler chickens is shown in Fig. 9. The treatment (F(4,149)=0.43; *p* = 0.788), sex (F(1,149)=0.12; *p* = 0.726) and their interaction (F(4,149)=0.57; *p* = 0.684) had no significant effect on the TI duration (*p* > 0.05). Average TI duration in female birds ranged from 171.2 sec (FL) to 284.3 sec (1/3SF), while in males, the lowest duration was 174.7 sec in the 1/3SF group and the highest duration was 311.5 sec in the FL group.

**Fig. 10:**
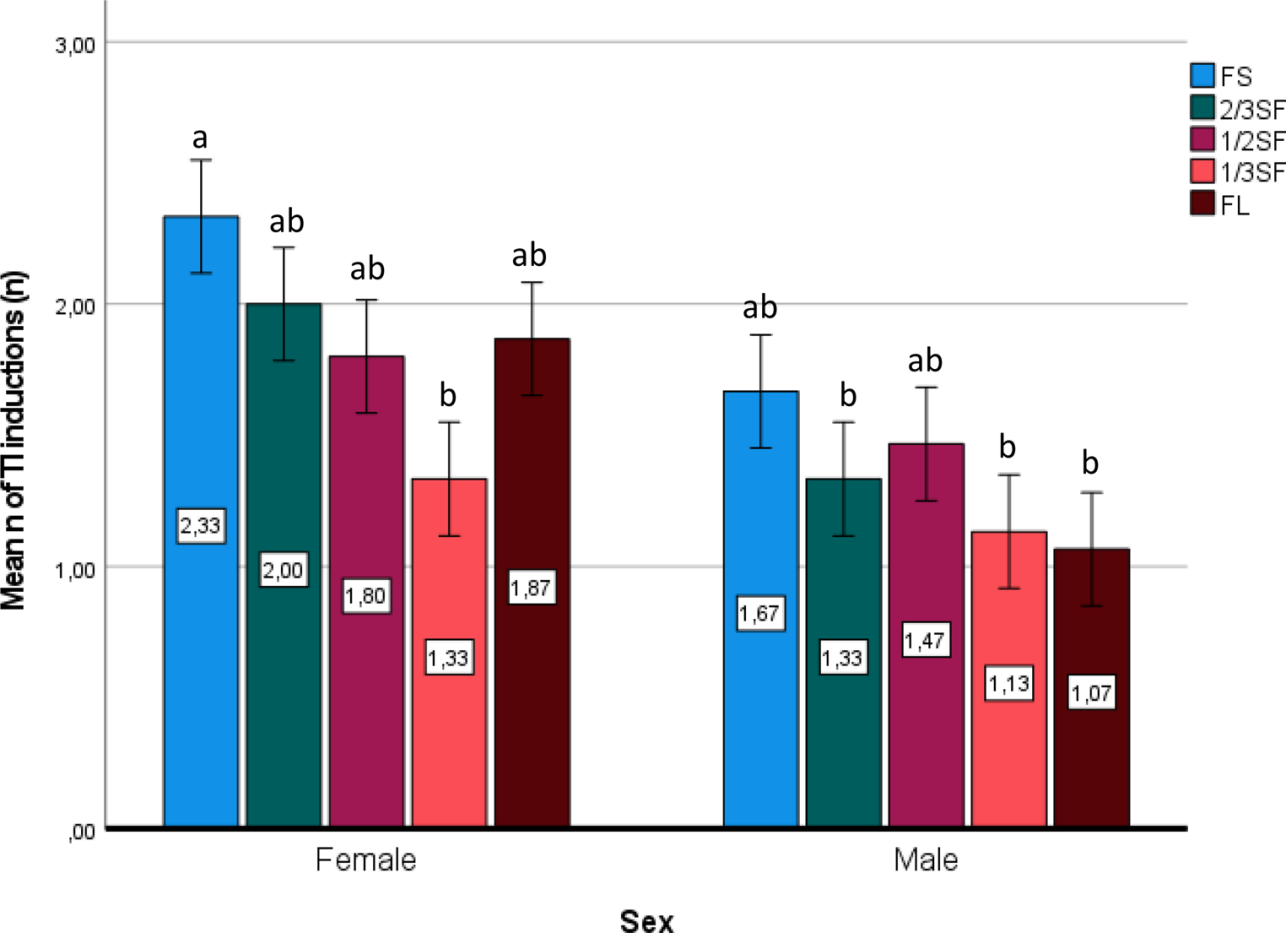
The effects of different flooring systems on the number of TI inductions in broilers. *Kruskal-Wallis χ2 = 9.575; df = 4; p*_*(treatment)*_* = 0.048; Kruskal-Wallis χ2 = 18.817; df = 1; p*_*(sex)*_* < 0.001; Kruskal-Wallis χ2 = 31.842; df = 9; p*_*(treatment × sex)*_* < 0.001.* FS: fully slatted system, 2/3SF: 2/3 slatted + 1/3 littered system, 1/2SF: 1/2 slatted + 1/2 littered system, 1/3SF: 1/3 slatted + 2/3 littered system, FL: fully littered system. ^a-b^: means indicated by different letters are significantly different based on the Tukey-HSD test results at the *p* < 0.05 level

The effects of different floor designs on the number of TI inductions in broilers are shown in Fig. 10. Treatment (χ^2^ = 9.575; *p* = 0.048), sex (χ^2^ = 18.817; *p* < 0.001), and their interaction (χ^2^ = 31.842; *p* < 0.001) had significant effects on floor designs. It is noteworthy that the average number of TI inductions in male broilers remained at lower levels compared to females. The female FS group (2.33) showed the highest induction number among all combinations. In contrast, the lowest TI inductions were recorded in the male FL (1.07) and male 1/3SF (1.13) groups, respectively, while among female birds, the lowest value was observed in the 1/3SF group with an average of 1.33.

*Behavioral Parameters.* Different floor designs resulted in significant differences in the passive, active, and comfort behaviors of the chickens (*p* < 0.01). Passive behaviors were highest in birds reared in the 1/3SF group (73.95%), followed by the other slatted floor (SF) groups, and lowest in the fully littered (FL) group (67.67%). Active behaviors were highest in the full litter system (25.91%), while the lowest values were determined in the full slatted system (23.05%). Comfort behaviors were highest in the FS and FL groups, respectively, followed by other groups with different slatted combinations (*p* < 0.001, Table 3).

**Table 3:** The effects of different floor designs on the main behavioral categories (passive, active, comfort) in broiler chickens (%)

Category	FS	2/3SF	1/2SF	1/3SF	FL	SEM	Treatment effect
Passive	69.98^b^	69.69^b^	69.59^b^	73.95^a^	67.67^c^	0.511	F(4,400)=21.291, *p* < 0.001
Active	23.05^b^	25.29^a^	24.82^ab^	20.57^c^	25.91^a^	0.494	F(4,400)=17.908, *p* < 0.001
Comfort	6.97^a^	5.01^c^	5.58^c^	5.47^c^	6.40^b^	0.179	F(4,400)=18.650, *p* < 0.001

**FS:**Fully slatted system, 2/3SF: 2/3 slatted + 1/3 littered system, 1/2SF: 1/2 slatted + 1/2 littered system, 1/3SF: 1/3 slatted + 2/3 littered system, FL: Fully littered system.

^**a,b,c**^**:**The means in the same row followed by different letters are significantly different according to Tukey’s test (*p* < 0.05).

**SEM: **Standard error of the mean

The frequency of different behavioral traits across treatment groups is shown in Table 4. Among passive behaviors, resting behavior was highest in the 1/3SF group (51.73%) and lowest in the FL group (46.49%) (*p* < 0.01). Sleeping behavior was highest in the FS group (22.15%), while a lower percentage of resting behavior was observed in the FL group (20.44%) (*p* < 0.05).

**Table 4:** The effects of different floor designs on the behavioral traits in broiler chickens (%)

Behavioral traits	FS	2/3SF	1/2SF	1/3SF	FL	SEM	Treatment effect
Resting	46.98^c^	49.49^b^	48.24^bc^	51.73^a^	46.49^cd^	0.530	F(4,400)=13.654, *p* < 0.01
Sleeping	22.15^a^	19.19^b^	19.98^b^	20.95^ab^	20.44^ab^	0.572	F(4,400)=3.381, *p* = 0.01
Standing	0.82^b^	0.99^b^	1.35^a^	1.27^a^	0.74^b^	0.097	F(4,400)=7.506, *p* < 0.01
Locomotion	0.88^b^	1.24^ab^	1.40^a^	1.23^ab^	1.41^a^	0.109	F(4,400)=3.415, *p* = 0.01
Feather pecking^1^	0.14	0.13	0.13	0.20	0.23	0.040	F(4,400)=1.794, *p* = 0.13
Aggressiveness^1^							
Escape^1^							
Exploratory	0.93^a^	0.41^bc^	0.57^b^	0.60^b^	0.31^c^	0.070	F(4,400)=10.145, *p* < 0.01
Foraging	0.00^c^	1.38^b^	1.94^a^	1.78^a^	2.11^a^	0.136	F(4,400)=39.236, *p* < 0.01
Eating	11.12^a^	10.74^a^	9.51^b^	9.11^b^	10.57^a^	0.276	F(4,400)=9.055, *p* < 0.01
Drinking	9.83^b^	11.33^a^	11.08^a^	7.59^c^	11.26^a^	0.268	F(4,400)=16.964, *p* < 0.01
Preening	6.22^a^	4.20^c^	4.65^b^	4.74^b^	5.64^b^	0.165	F(4,400)=26.201, *p* < 0.01
Stretching	0.72	0.67	0.81	0.58	0.64	0.071	F(4,400)=1.248, *p* = 0.290
Dust bathing	0.00^b^	0.11^a^	0.10^a^	0.13^a^	0.11^a^	0.030	F(4,400)=3.724, *p* = 0.05

**FS:**Fully slatted system, 2/3SF: 2/3 slatted + 1/3 littered system, 1/2SF: 1/2 slatted + 1/2 littered system, 1/3SF: 1/3 slatted + 2/3 littered system, FL: Fully littered system.

^1^ Given as the total frequency of feather pecking + aggressiveness + escape behaviors.

^**a-d**^**:**The means in the same row followed by different letters are significantly different according to Tukey’s test (*p* < 0.05).

**SEM:**Standard error of the mean

Among the active behavioral traits, standing was highest in the 1/3SF group, while lower results were found in the FS (0.82%), 2/3SF (0.99%), and FL (0.74%) groups (*p* < 0.01).

The highest locomotion behavior was observed in the FL group (1.41%), followed by the other slatted systems, while the lowest values were found in the SF group (0.88%) (*p* < 0.01). Aggressive, feather-pecking, and escape behaviors were higher on FL (0.23%) system, but differences between treatments were not significant. Exploratory behavior was highest in the FS group (0.93%) and lowest in the FL group (0.31%) (*p* < 0.01). The highest foraging behavior was observed in the FL group (2.11%), and this level decreased as the level of slats increased (*p* < 0.01). Eating behavior was the highest in the FS (11.12%), 2/3SF (10.74%), and FL (10.57%) groups (*p* < 0.01). This behavior could not be determined in the FS system. Water drinking behavior was higher in the 2/3SF, FL, and 1/2SF groups (11.33%, 11.26%, and 11.08%, respectively) (*p* < 0.01).

Among comfort behaviors, feather preening was the highest in the FS group (6.22%), while the lowest value was observed in the 2/3SF group (*p* < 0.01). Stretching behavior was highest in the 1/2SF group (0.72%), but differences between groups were not significant. Dust bathing was similar in all groups with littered systems, and no significant differences were found between treatments.

## Discussion

Previous studies highlight that while fully littered systems allow for the expression of natural behaviors such as foraging and dust bathing in broilers, they often pose risks for contact dermatitis and welfare issues due to high litter moisture (Mayne 2005; Da Costa et al. 2014; Mounir et al. 2025). Conversely, although fully or partially slatted floors effectively separate birds from manure and improve hygiene (Topal 2021; May et al. 2024), they are known to restrict natural activities and negatively impact leg health and walking ability (Blokhuis 1989; Shields and Greger 2013; Baxter et al. 2018). Therefore, this study aimed to investigate whether partially slatted flooring combinations could serve as a compromise to mitigate the welfare and behavioral trade-offs typically associated with these extreme housing systems.

*Welfare traits.* Animal welfare is a complex concept that covers the physical and emotional state of an animal, its ability to cope with its environment, and its quality of life (Jacobs et al. 2023). The welfare of chickens is largely dependent on the rearing system. Foot and leg problems and contact dermatitis are frequently observed welfare-related issues in fast-growing broilers (Weeks et al. 2000; Bokkers and Koene 2004; Arnould et al. 2011). In this study, no FPD occurred in the treatment groups at a level that could be scored. We suggest that, in addition to the slatted flooring, the effective maintenance of litter quality and low litter moisture levels contributed to lack of FPD (Da Costa et al. 2014; Sarıca et al. 2014; Boz et al. 2017; Sonnabend et al. 2022). Almeida et al. (2018) reported lower FPD incidence on plastic slatted floors compared to littered floors.

In the study, as the slatted area increased, there was also an increase in occurence of hock burns. Ghayas et al. (2021) stated that rearing systems have a significant effect on FPD and hock burns, reporting an increase in both lesions as the flooring hardened and moisture increased. Similar to the results of our study, Çavuşoğlu and Petek (2019) stated that incidence of FPD and hock burns were higher on slatted floors compared to littered systems. Dikmen and Gündüz (2025) reported higher occurrence of hock burns (1.30 and 1.20) and FPD (2.60 and 0.95) compared to cage flooring systems. Although slatted floors improve hygiene by minimizing fecal contact (Almeida et al. 2017; Li et al. 2017), they introduce severe physical stressors. The increased hock burn severity and impaired walking ability on slatted floors result from the continuous pressure of hard plastic on the joints, exacerbated by the extended resting periods of fast-growing broilers (Weeks et al. 2000; Ghayas et al. 2021). Moreover, partially slatted systems fail to provide an adequate compromise. Even with a high litter ratio, male broilers exhibited significantly poorer leg health than those on fully littered floors, confirming that access to a yielding substrate is crucial for joint health and walking ability (Fouad et al. 2008; Almeida et al. 2018). Additionally, reduced litter area restricts simultaneous natural behaviors like dust bathing (Baxter et al. 2018). Consequently, partially slatted floors do not resolve the hygiene-welfare conflict but merely shift the trade-off from contact dermatitis to physically induced joint lesions and behavioral restriction.

In our study, it was observed that as the use of slatted flooring decreased, breast skin burn/discoloration decreased. Supporting the findings of our study, Li et al. (2017) reported higher incidence of breast burns in broilers reared on sawdust littered compared to those reared on slatted flooring. Almeida et al. (2017) reported no breast burning in broilers reared on litter and slatted floors under appropriate conditions without heat stress. They also reported that breast skin burns increased in broilers under conditions of heat stress. In another study, Almeida et al. (2018) reported that breast burns were not observed in broilers reared on litter, while mild breast burns were observed in male broilers reared on slatted floors.

In welfare-focused housing systems, litter management is one of the most important criteria for animal welfare. Litter quality is used as an indicator of poor welfare in broilers (Dawkins et al. 2004; Haslam et al. 2007; Knowles et al. 2008).

De Jong et al. (2014) reported that poor litter quality causes quick contamination of feathers, reducing feather cleanliness. Ghayas et al. (2021) found that rearing systems have a significant effect on feather cleanliness condition score.

They also noted that fast-growing broilers reared in intensive systems had lower feather cleanliness condition than slow-growing ones, while fast-growing genotypes had higher feather condition in free-range rearing systems. Supporting the findings of our study, Çavuşoğlu and Petek (2019) reported that the feathers of broiler chickens reared on slatted floors were significantly cleaner than those reared in littered systems.

Broilers on slatted floors have better cleanliness conditions because they have very little contact with feces (Almeida et al. 2017; Li et al. 2017). These findings, which are largely consistent with the outcomes of our study, are seen as positive aspects of using slatted floors for broilers.

The walking score, which is a good indicator of lameness in determining foot and leg problems, is widely used to determine the welfare status of broiler chickens. It has been reported that walking ability deteriorates with an increase in FPD lesions and hock joint lesions (Son 2013). In particular, broilers spend most of their time close to slaughter in a sitting or lying position, and therefore their walking ability is reported to deteriorate (Weeks et al. 2000).

Caplen et al. (2012) reported that broiler welfare is compromised when walking ability is moderate or severely impaired. In our study, which used plastic flooring at different levels, we found that walking ability was significantly reduced in male broilers reared on slatted floor system. Çavuşoğlu and Petek (2019), similar to the findings of this study, stated that walking ability decreased with increased weight gain and that slow-growing genotypes had better walking ability. Li et al. (2017) and Sánchez-Casanova et al. (2020) reported that fast-growing broiler chickens at six weeks of age had poorer walking ability on slatted floors. Fouad et al. (2008) also found that chickens reared on littered systems had better walking ability than those reared in cages. They also reported that exercise and activity limitations due to confined space contribute to the prevalence of walking problems in caged chickens and negatively affect welfare conditions (Reiter and Bessei 1998). Almeida et al. (2018) found that male chickens reared on sawdust litter had good walking ability.

In our study, the effect of different levels of slatted and littered floor designs on the TI duration in broilers was insignificant, while some studies have reported differences between different rearing systems and genotypes. Our study results are consistent with the study by Çavuşoğlu and Petek (2019), which reported that different flooring systems did not have a significant effect on the TI duration. Ghayas et al. (2021) reported that broiler chickens reared in a free-range system had a shorter TI duration than those reared in an intensive system. The same study also observed significant differences in TI duration between fast- and slow-growing genotypes. Fast-growing genotypes reared in an intensive system recorded a higher TI duration than slow-growing ones. Similarly, it was reported that fast-growing genotypes in the free-range system also had a higher TI duration than slow-growing ones. Abdourhamane and Petek (2024) found that TI durations were longer in slow-growing chickens (121.34 sec) than in fast-growing chickens (78.52 sec) but reported that there was no significant difference.

*Behavioral traits.* Our study is consistent with the findings of Çavdarcı et al. (2022), who reported that birds on FL system exhibited lower resting behavior (20.4%) compared to those on SF system. The high percentage of active behavior on FL system indicates that active behaviors occur instead of passive behaviors such as resting and sleeping. Bokkers and Koene (2003) reported that in fast-growing broilers, increased growth rate significantly affects feeding, standing, and resting, as well as reduced locomotion behavior. Bach et al. (2019) reported that broiler chickens exhibited 30% active, 65% passive, and 5% comfort behaviors. Although these results are consistent with our study regarding comfort behaviors, in our study, more active and less passive behaviors were determined in all floor systems.

The increased occurrence of natural behaviors in broiler chickens reared on littered systems is considered an advantage. Access to litter material has a significant effect on the natural behavior and physiology of birds (Cabrera et al. 2018). In the non-littered (i.e. slatted) systems, the absence of litter material restricts some natural behaviors (e.g., dust bathing, foraging, preening) (Blokhuis 1989). Previous studies have shown that most natural behaviors in broilers, such as scratching, foraging, stretching, and dust bathing, are exhibited when contact with litter is provided (Shields et al. 2005; Villagra et al. 2014; Baxter et al. 2018). Our study found that the percentage of resting and sleeping behavior was higher than the percentage of other behaviors. Li et al. (2017) reported that broiler chickens reared on FS system spent longer periods resting than chickens reared on FL systems because of the air circulation under the slat material. Ghayas et al. (2021) observed resting behavior in 60% of fast- and slow-growing broilers in the intensive system and in 45% in the free-range system. The percentage of locomotion behavior was 11.25% in the intensive system and 25.60% in the free-range system. Fouad et al. (2008) found resting behavior to be 14.71% and 11.67% in littered and cage systems, respectively. Similarly, the percentage of resting behavior was higher on littered floors (60.7%) than in cage systems (56.7%). Wilhelmsson et al. (2019) reported that fast-growing broilers exhibited less foraging behavior than slow-growing ones because they spent more time resting and less time walking around.

It has been reported that FS systems without litter on the ground do not allow chickens to perform behaviors such as scratching and foraging, so these behaviors are replaced by exploratory and feeding behaviors (Blokhuis 1989). Consistent with this study, our study found that eating and exploratory behaviors were higher in the FS system. Çavdarcı et al. (2022) stated that exploratory behavior was the highest in FS system in broiler chicks at an early age. In our study, eating behavior was higher in the FS system, with a behavior percentage of 11.12% compared to other floor designs.

The percentage of feather pecking, aggressiveness and escape behavior was higher in the FL system, and it was observed that the percentage of these behaviors decreased as the littered area decreased. Supporting these results, Fortomaris et al. (2007) reported that aggressive and wing-flapping behavior in broiler chickens was higher in the littered systems compared to the cage system. Abdourhamane (2019) stated that aggressive and wing-flapping behavior was higher in the littered system. In our study, similar results were found in littered+slatted combinations. Since dust bathing is not possible on a FS system, it is thought that preening behavior may be exhibited instead.

Li et al. (2017) reported that feather preening behavior improved in broilers reared on a SF systems because they had no contact with the litter material. Dust bathing, puffing, and preening behaviors are good indicators of the welfare and health condition of broilers. During the dust bathing and scratching, feathers come into contact with the litter. If litter management is poor, feathers may become dirty, so the cleanliness of feathers reflects litter and floor conditions. Çavuşoğlu and Petek (2019) reported that slatted floor systems had an effect on feather preening, standing, and resting behavior over 4 weeks in broilers.

## Conclusions

This study revealed a clear contradiction between physical and behavioral welfare indicators and hygiene parameters depending on the floor design in broiler production. While fully or partially slatted floor systems significantly improved body cleanliness and breast feather condition by minimizing contact with manure, they had detrimental effects on physical welfare. Specifically, slatted systems resulted in increased hock burn scores, breast skin discoloration, and impaired walking ability, particularly in male chickens. Furthermore, the absence or reduction of litter material in slatted and combined systems restricted the expression of basic natural behaviors such as foraging and dust bathing, leading instead to an increase in passive behaviors and preening frequency. Although partially slatted (combined) systems offered intermediate results, they did not completely eliminate the negative effects of slats on leg health and behavioral flexibility, meaning they fell short of providing an ideal compromise between hygiene and welfare. Ultimately, while slatted floors offer advantages in terms of hygiene management, fully-littered systems with optimized bedding quality remain the most suitable approach for supporting leg health and allowing the expression of natural behaviors in fast-growing broilers. Future studies should focus on innovative slatted materials or designs that minimize physical injuries while maintaining hygiene advantages to ensure the sustainability of alternative housing systems.

## Data Availability

Not applicable.

## Code availability

Data were analyzed using SPSS (Version 21.0).
